# Supplementary material for: Interleukin-8 as a candidate for thymoma identification and recurrence surveillance
Source: Nat Commun. 2020 Sep 28;11:4881. doi: 10.1038/s41467-020-18697-x (PMC7522267; doi:10.1038/s41467-020-18697-x)
Supplement: Supplementary file 1 — Supplementary Information [file 41467_2020_18697_MOESM1_ESM.pdf]

Supplementary Information

Interleukin-8 as a candidate for thymoma identification and recurrence surveillance

Gao et al.

***Corresponding Authors:**

Jianyong Ding, E-mail: ding.jianyong@zs-hospital.sh.cn

Haikun Wang, E-mail: hkwang@ips.ac.cn

Supplementary Table 1. Antibodies used in this study

Antigen	Fluorophor	Clone Number	Catalogue Number	Provider	Dilution
CD15	eFluor 450	MMA	43-0158-42	eBioscience	1:30
TCRα β	APC	IP26	17-9986-42	eBioscience	1:60
CD3	Alexa Fluor 700	UCHT	557943	Biolegend	1:60
CD45RA	Brilliant Violet 785	HI100	304140	Biolegend	1:100
CCR7	PE-Cy7	G043H7	353226	Biolegend	1:60
IL-8	FITC	E8N1	554720	Biolegend	1:30
CD31	PerCP/eFluor 710	UVM59	46-0319-42	Invitrogen	1:60
CD14	APC-Cy7	M5E2	301819	Biolegend	1:60
CD8	Brilliant Violet 650	RPA-T8	301041	Biolegend	1:150
CD4	Brilliant Violet 605	SK3	63-0047-42	eBioscience	1:60
CR2	PE	BU32	354903	Biolegend	1:30
PTK7	--	Poly-clonal		*AbMART	1:300

* Rabbit anti-PTK7 antibody was generated by AbMART Co.

Supplementary Table 2. Sequences of primers and probes for sjTrec quantification

Gene	Primer name	Sequence
TRECs	forward	5’-CAC ATC CCT TTC AAC CAT GCT-3’
	reverse	5’-TGC AGG TGC CTA TGC ATC A-3’
	probe	5’-FAM-ACA CCT CTG GTT TTT GTA AAG GTG CCC ACT-TAMRA-3’
TCRACs	forward	5’-TGG CCT AAC CCT GAT CCT CTT-3’
	reverse	5’-GGA TTT AGA GTC TCT CAG CTG GTA GTA CAC-3’
	probe	5’-FAM-TCC CAC AGA TAT CCA GAA CCC TGA CCC-TAMRA-3’

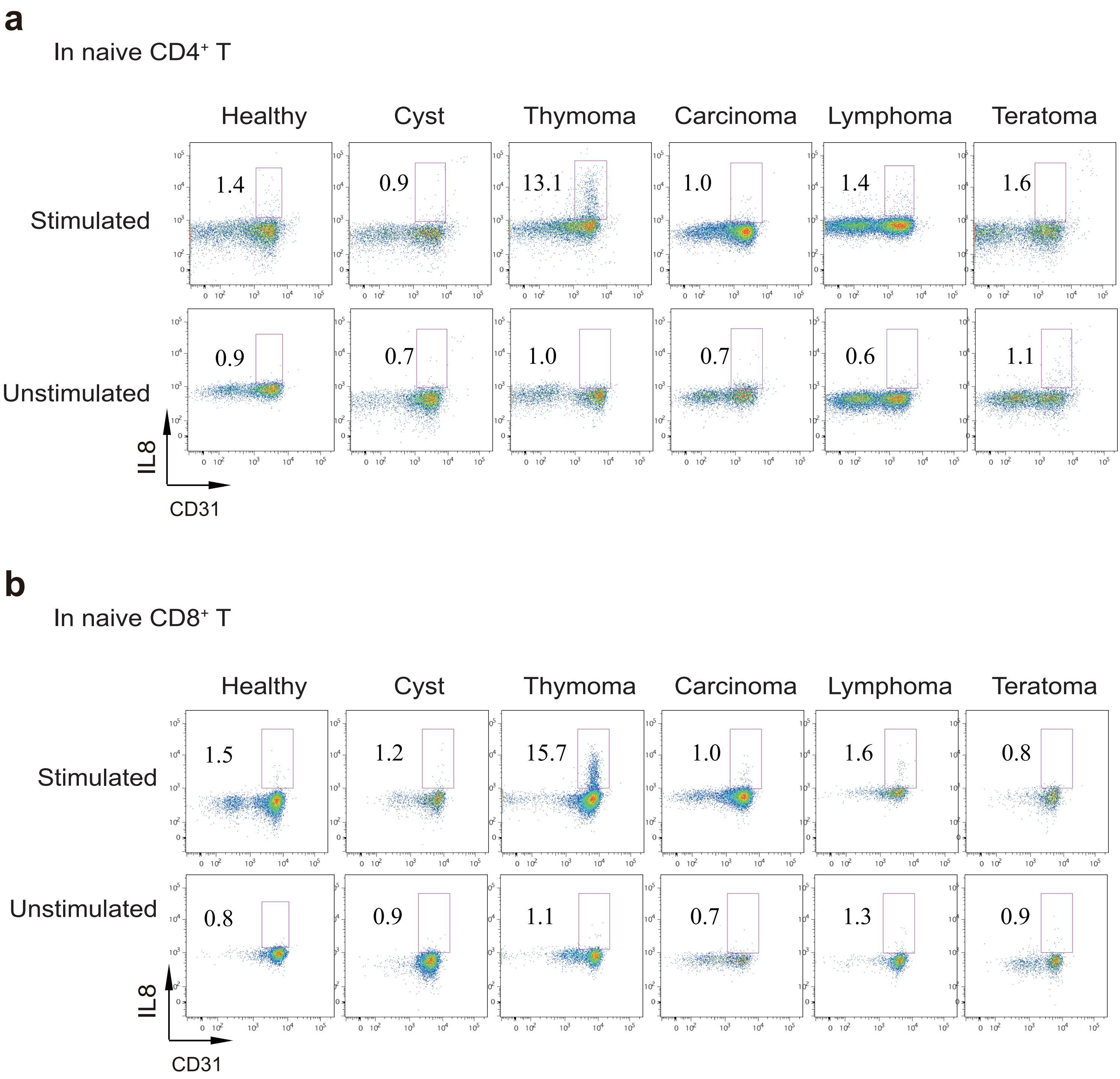

Supplementary Figure 1. Representative flow cytometry plots of IL-8$^+$ naïve T cells in patients in the discovery set with different thymic masses. (a) Representative flow cytometry plots of IL-8$^+$CD31$^+$ naïve CD4$^+$ T cells in PMA-stimulated or unstimulated PBMCs from patients with thymic tumors and age-matched healthy controls. (b) Representative flow cytometry plots of IL-8$^+$CD31$^+$ naïve CD8$^+$ T cells in PMA-stimulated or unstimulated PBMCs from patients with thymic tumors and age-matched healthy controls.

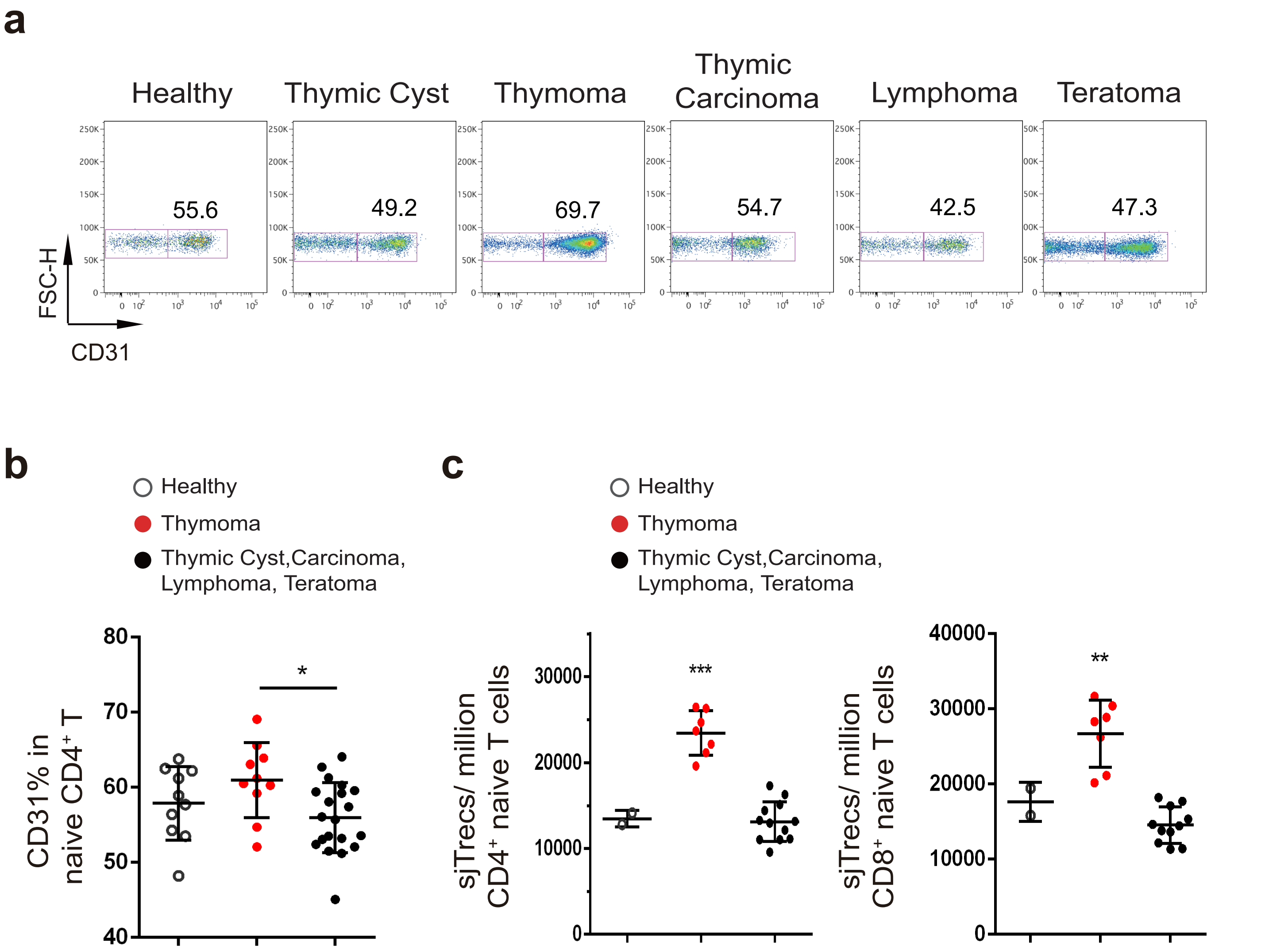

Supplementary Figure 2. The proportions of $CD31^+$ RTEs and sjTrecs contents in naïve T cells are increased in patients with thymoma. (a-b) CD31 expression were analyzed by flow cytometry in $CD4^+$ naïve T cells ($CD235a^-CD19^-CD14^-CD3^+TCR\alpha\beta^+CD4^+CD8^-CD45RA^+CCR7^+CD31^+$) in peripheral blood mononuclear cells (PBMCs) from patients in the discovery set with different thymic tumors (thymomas, thymic cysts, thymic carcinomas, lymphomas and teratomas) and age-matched healthy controls. (a) Representative flow cytometry plots of $CD31^+$ cells in naïve $CD4^+$ T cells in patients with thymic tumors. (b) The proportions of $CD31^+$ cells in naïve $CD4^+$ T cells in patients and age-matched healthy controls in the discovery set. (c) sjTrecs contents were analyzed in sorted naïve T cells in patients with thymic masses and age-matched healthy controls. Left panel, sjTrecs contents in $CD4^+$ naïve T cells in patients with thymic masses and age-matched healthy controls; Right panel, sjTrecs contents in $CD8^+$ naïve T cells in patients with thymic masses and age-matched healthy controls. Data in (b-c) are shown as the mean ± standard deviation (SD). Statistical differences in (b) were determined by two-sided Mann-Whitney test and adjusted with the Benjamini–Hochberg procedure. Adjusted P values were indicated by * ($p<0.05$). Statistical differences in (c) were determined by two-sided Krusal-Wallis Analysis of Variance test and adjusted with the Benjamini–Hochberg procedure. Adjusted P values were indicated by **($p<0.01$), or *** ($p<0.001$). Source data are provided as a Source Data file.

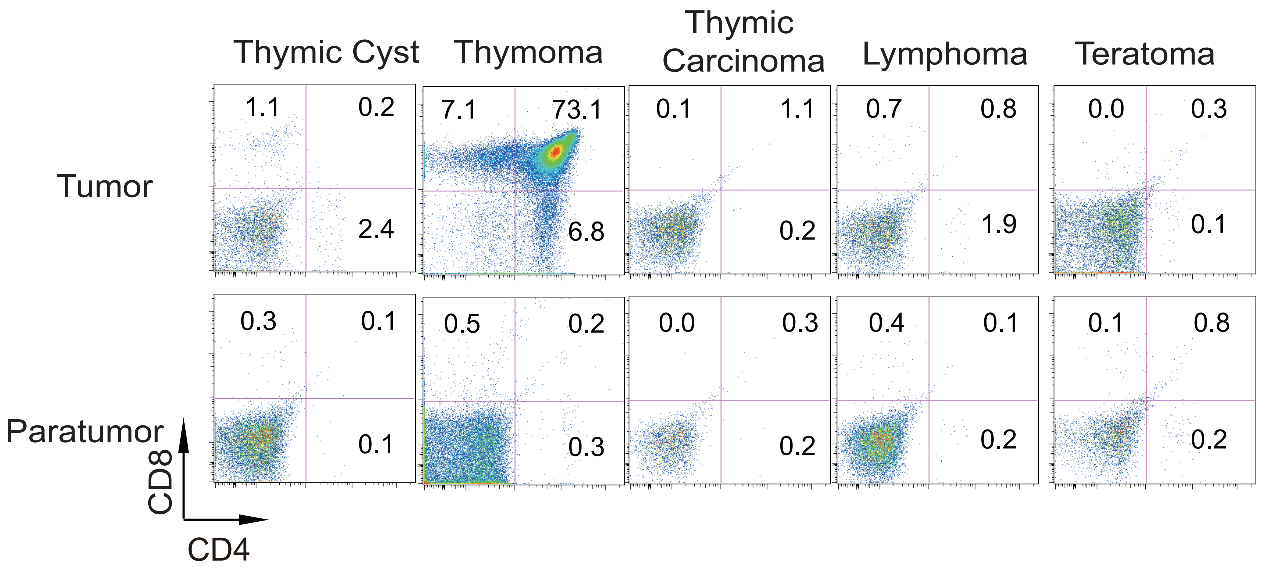

Supplementary Figure 3. Representative flow cytometry plots of $CD4^{-}CD8^{-}$ double negative, $CD4^{+}CD8^{+}$ double positive, $CD4^{-}CD8^{+}$ single positive and $CD4^{+}CD8^{-}$ single positive thymocytes in tumor and paratumor tissues from patients with different thymic tumors (thymomas, thymic cysts, thymic carcinomas, lymphomas and teratomas).

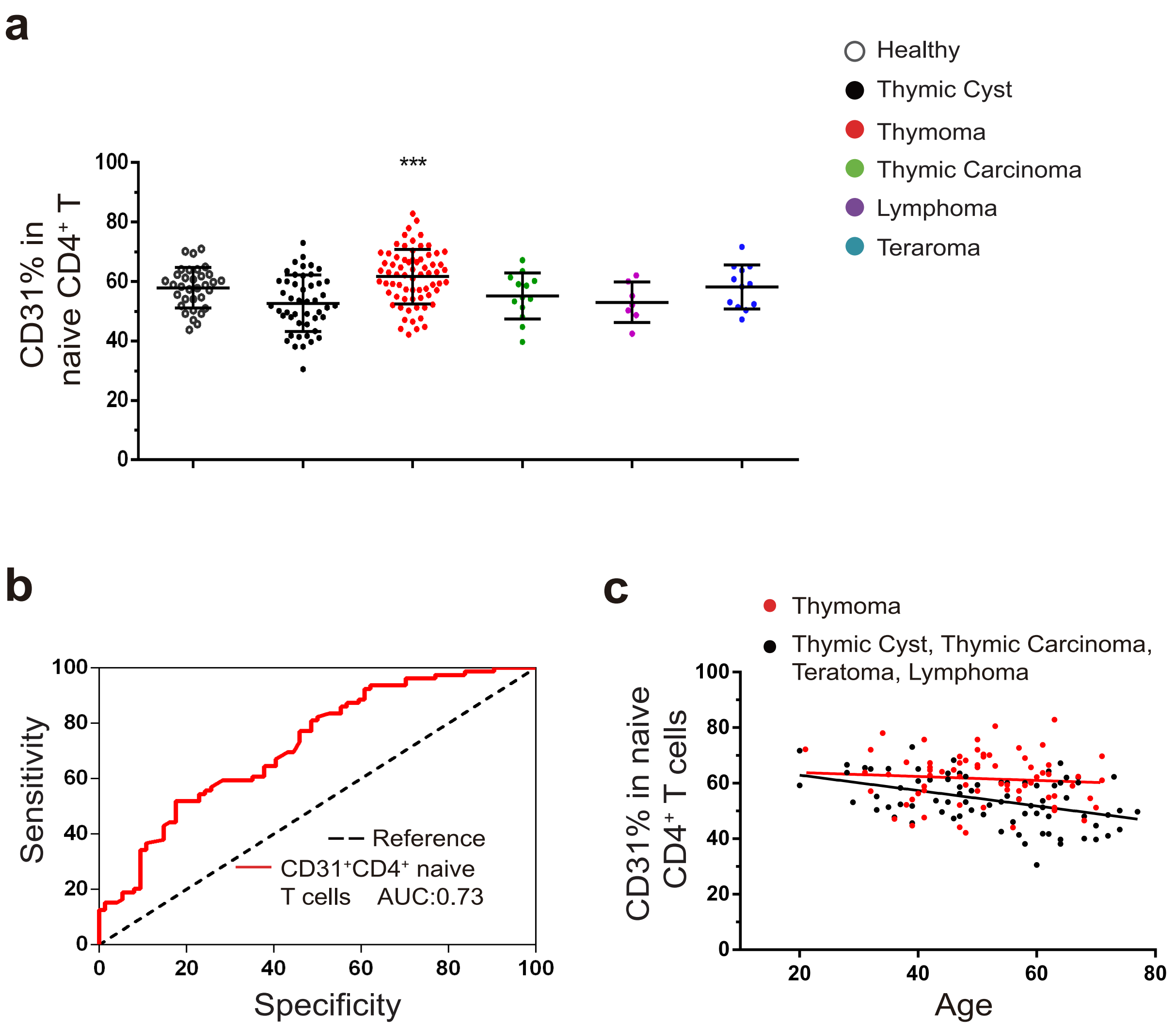

Supplementary Figure 4. CD31 evaluation can not distinguish thymomas from other thymic tumors accurately. (a) The proportions of $CD31^+$ cells in naïve $CD4^+$ T cells in patients and age-matched healthy controls in the validation set. (b) ROC curve analysis was performed to evaluate the diagnostic sensitivity and specificity of CD31 as a biomarker of thymomas. (c) The frequencies of $CD31^+CD4^+$ naïve T cells in patients with thymomas and other thymic tumors of various ages in the validation set. Data in (a) are shown as the mean ± standard deviation (SD). Statistical differences in (a) were determined by two-sided Krusal-Wallis Analysis of Variance test and adjusted with the Benjamini–Hochberg procedure. Adjusted P values were indicated by *** ($p<0.001$). Source data are provided as a Source Data file.

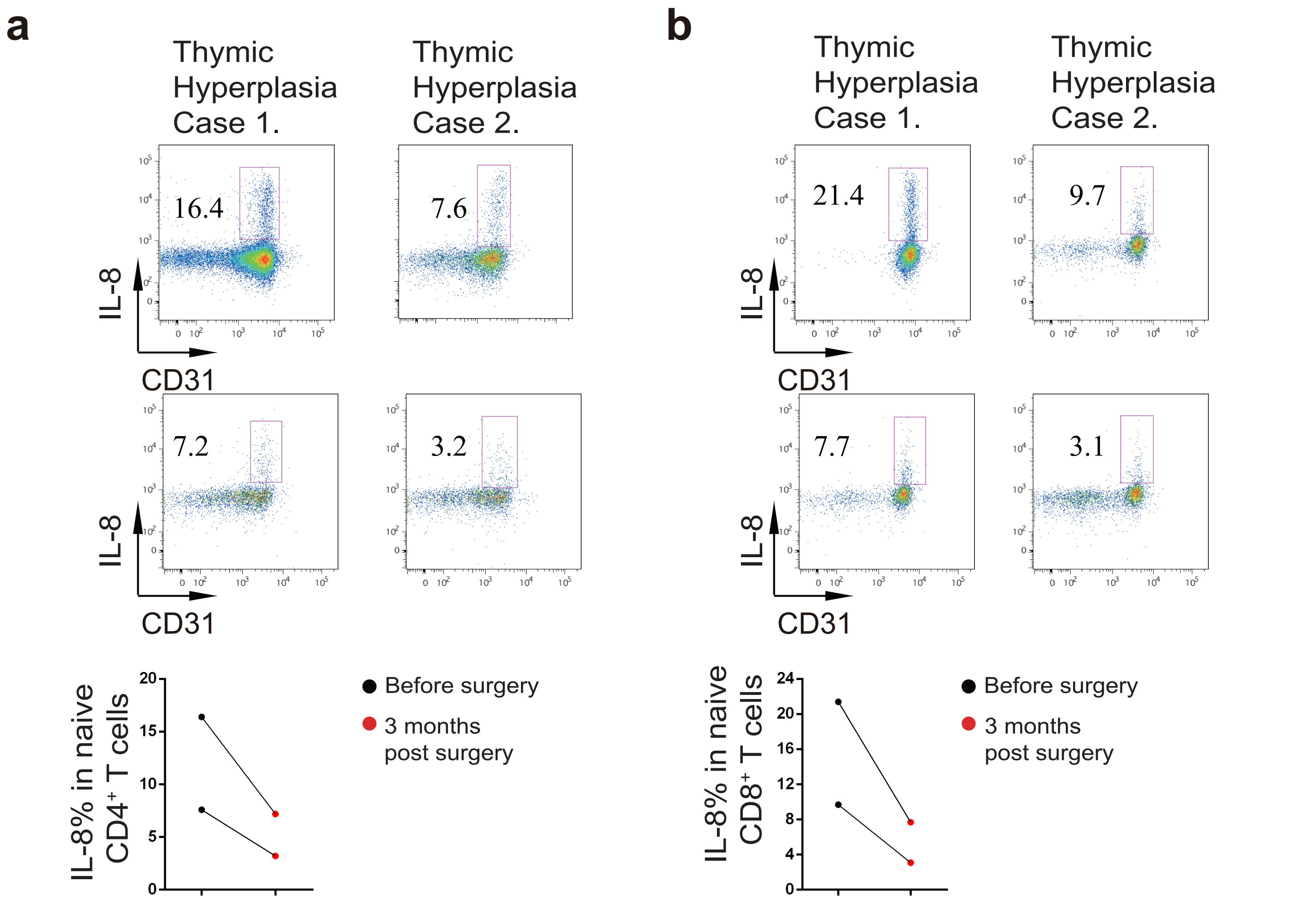

Supplementary Figure 5. IL-8^{+} naïve T cells are increased in patients with thymic hyperplasia. The proportions of IL-8^{+} naïve T cells in two representative patients with thymic hyperplasia were measured before and 3 months post thymic surgery. (a) Representative flow cytometry plots of IL-8^{+}CD31^{+} cells in CD4^{+} naïve T cells in PBMCs from patients with thymic hyperplasia before and after tumor removal (upper panel); summary of the frequencies of IL-8^{+}CD31^{+} cells in CD4^{+} naïve T cells in PBMCs from patients with thymic hyperplasia before and after tumor removal (lower panel). (b) Representative flow cytometry plots of IL-8^{+}CD31^{+} cells in CD8^{+} naïve T cells in PBMCs from patients with thymic hyperplasia before and after tumor removal (upper panel); summary of the frequencies of IL-8^{+}CD31^{+} cells in CD8^{+} naïve T cells in PBMCs from patients with thymic hyperplasia before and after tumor removal (lower panel). Numbers adjacent to the outlined areas of flow cytometry plots in (a-b) indicate the percentages of IL-8^{+} naïve T cells. Source data are provided as a Source Data file.

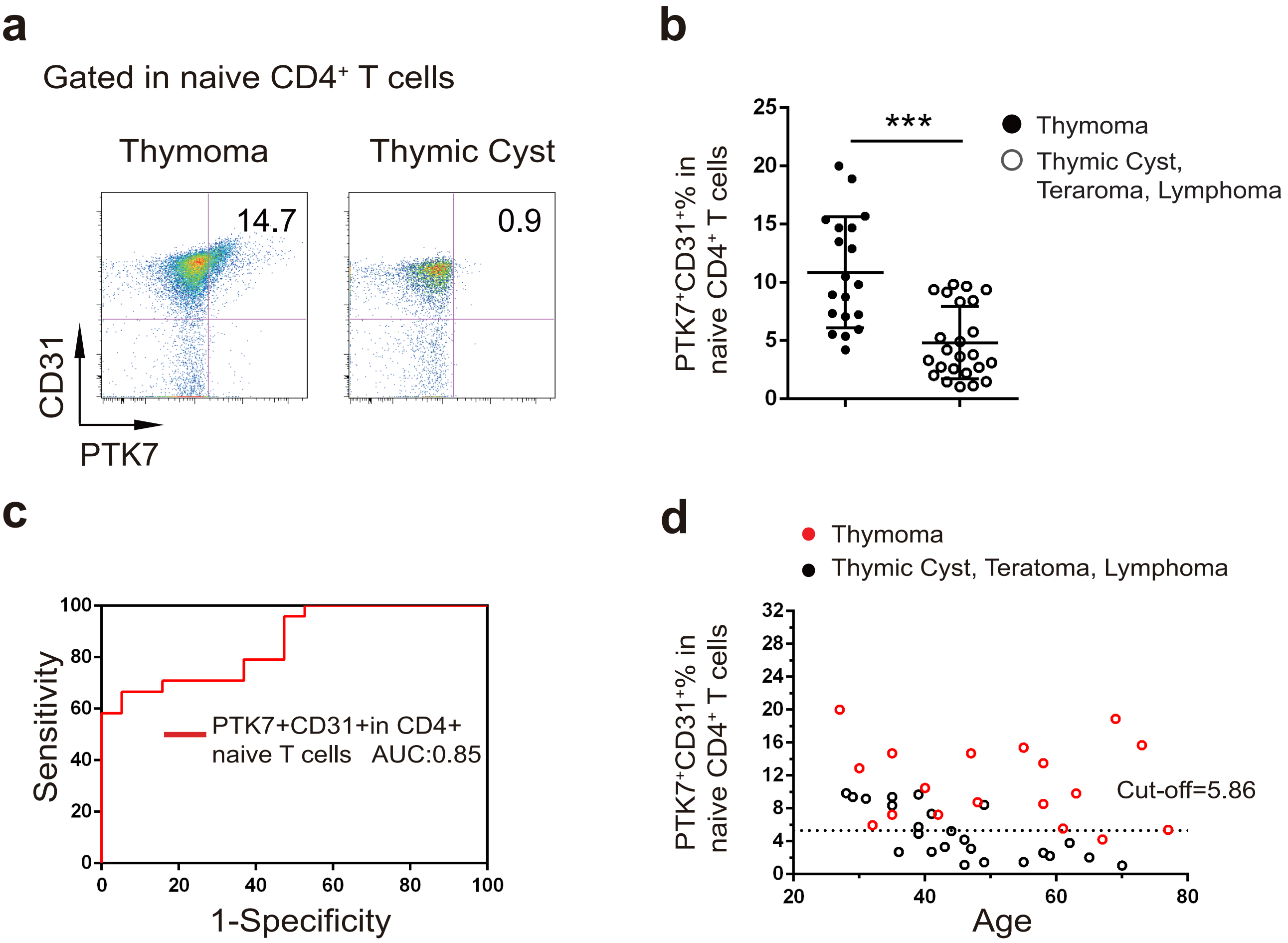

Supplementary Figure 6. The proportions of PTK7⁺ naïve T cells are increased in thymoma patients. PTK7⁺CD31⁺ cells within naïve T cells from patients with thymoma and other thymic masses were analyzed by flow cytometry. (a) Representative flow cytometry plot of PTK7⁺CD31⁺ naïve T cells in PBMCs from patients with thymoma and other thymic tumors. (b) Statistical data of PTK7⁺CD31⁺ naïve T cells in patients with thymoma and other thymic masses. (c) Diagnostic performance of PTK7⁺CD31⁺ naïve T cells in identifying patients with thymomas from patients with other thymic tumors was assessed by ROC curve analysis. (d) The frequencies of PTK7⁺CD31⁺ naïve T cells in patients with thymomas and other thymic tumors of various ages. The dashed line indicates the optimum cut-off point calculated by applying Youden's J statistic to ROC curves. Data in (b) are shown as the mean ± standard deviation (SD). Statistical differences were determined by two-sided Mann-Whinney test and adjusted with the Benjamini–Hochberg procedure. Adjusted P values were indicated by *** ($p<0.001$). Source data are provided as a Source Data file.

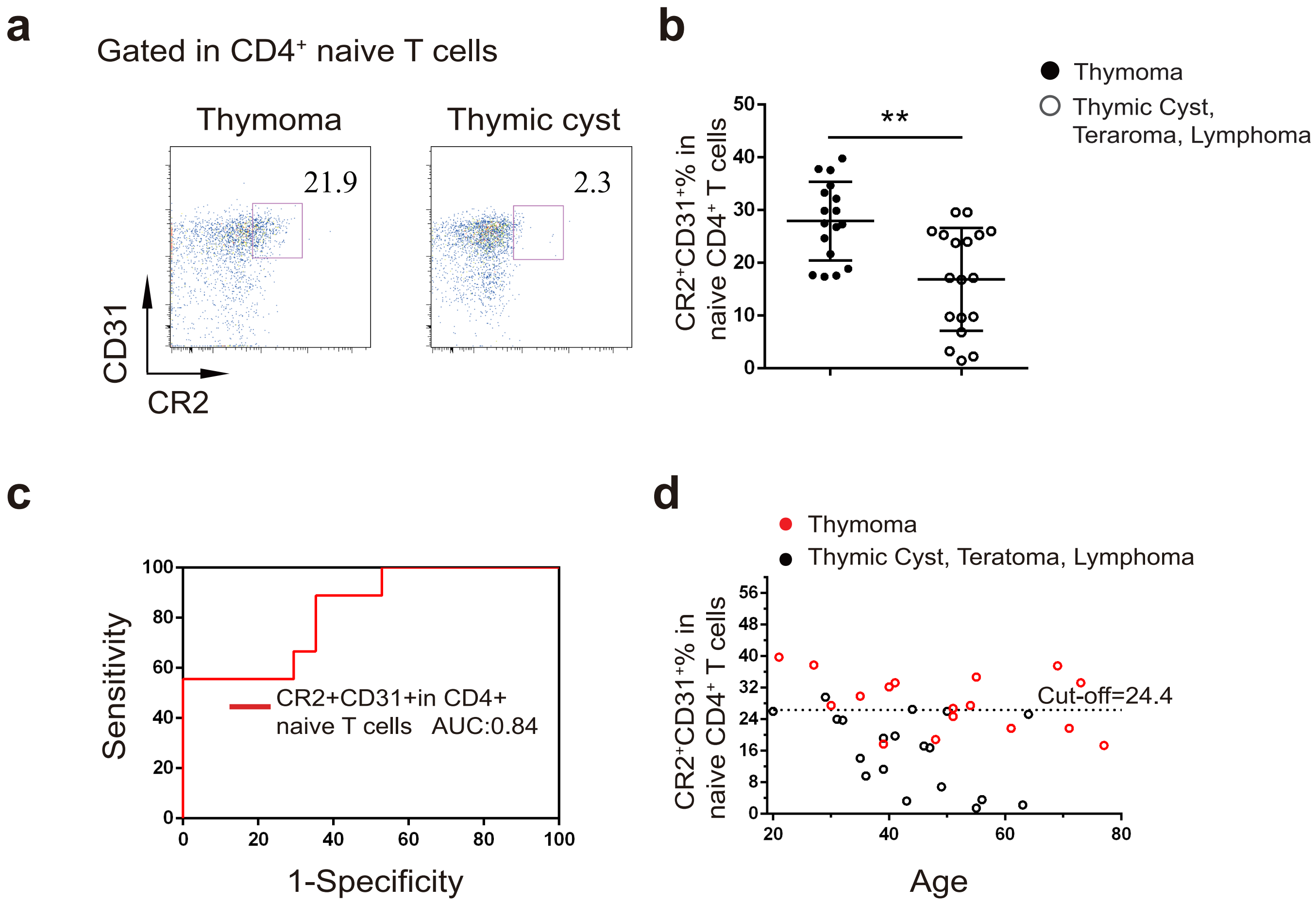

Supplementary Figure 7. The proportions of $CR2^+$ naïve T cells are increased in thymoma patients. $CR2^+CD31^+CD25^-$ naïve T cells from patients with thymoma and other thymic masses were analyzed by flow cytometry. (a) Representative flow cytometry plot of $CR2^+$ naïve T cells in PBMCs from patients with thymoma and other thymic masses. (b) Statistical data of $CR2^+$ naïve T cells in patients with thymoma and other thymic tumors. (c) Diagnostic performance of $CR2^+$ naïve T cells in identifying patients with thymomas from patients with other thymic tumors was assessed by ROC curve analysis. (d) The frequencies of $CR2^+$ naïve T cells in patients with thymomas and other thymic tumors of various ages. The dashed line indicates the optimum cut-off point calculated by applying Youden's J statistic to ROC curves. Data in (b) are shown as the mean ± standard deviation (SD). Statistical differences were determined by two-sided Mann-Whinney test and adjusted with the Benjamini–Hochberg procedure. Adjusted P values were indicated by ** ($p<0.01$). Source data are provided as a Source Data file.

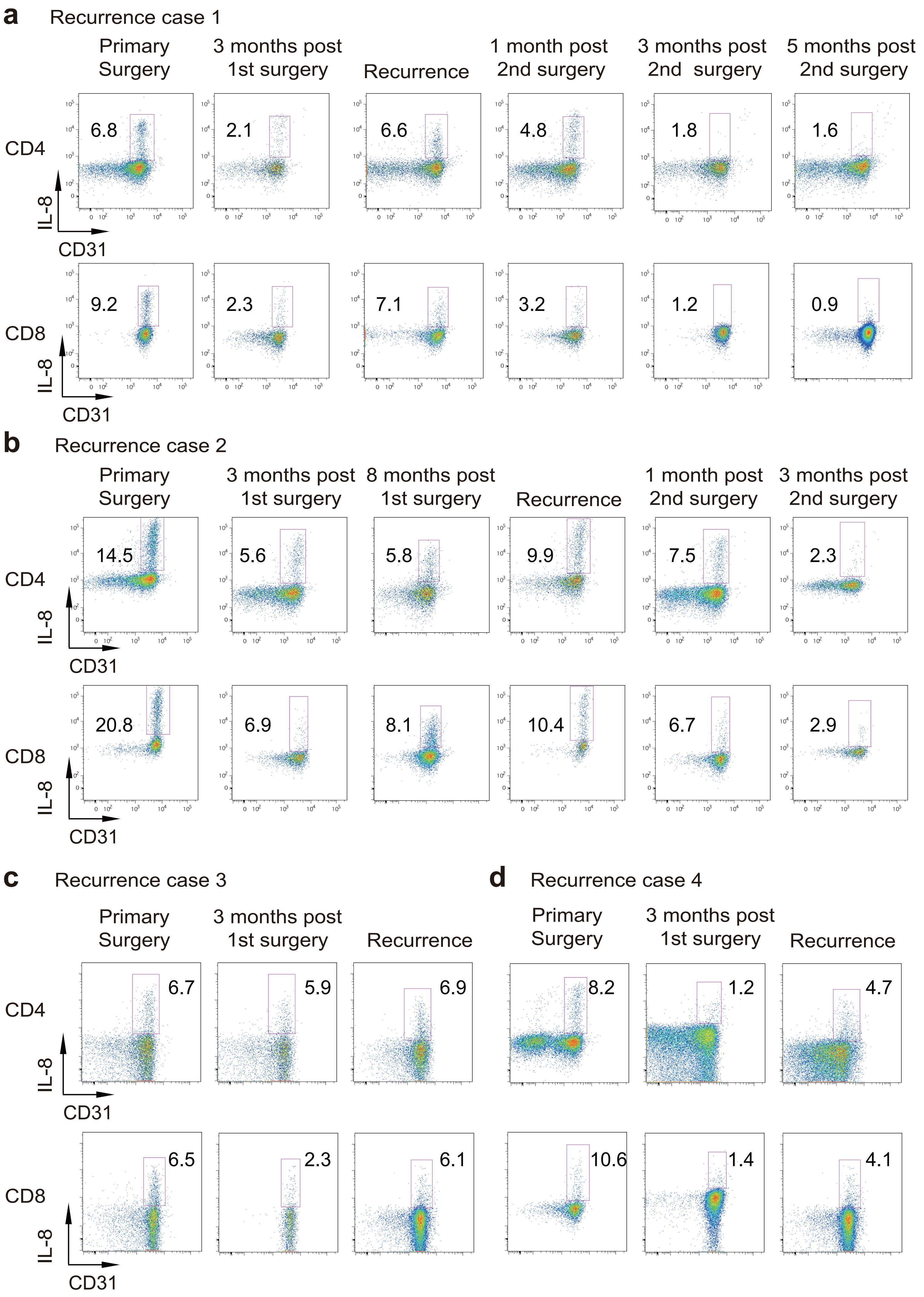
a
Recurrence case 1
Primary Surgery
3 months post 1st surgery
Recurrence
1 month post 2nd surgery
3 months post 2nd surgery
5 months post 2nd surgery
CD4
6.8
2.1
6.6
4.8
1.8
1.6
CD8
9.2
2.3
7.1
3.2
1.2
0.9
IL-8
CD31
b
Recurrence case 2
Primary Surgery
3 months post 1st surgery
8 months post 1st surgery
Recurrence
1 month post 2nd surgery
3 months post 2nd surgery
CD4
14.5
5.6
5.8
9.9
7.5
2.3
CD8
20.8
6.9
8.1
10.4
6.7
2.9
c
Recurrence case 3
Primary Surgery
3 months post 1st surgery
Recurrence
CD4
6.7
5.9
6.9
CD8
6.5
2.3
6.1
d
Recurrence case 4
Primary Surgery
3 months post 1st surgery
Recurrence
8.2
1.2
4.7
10.6
1.4
4.1

Supplementary Figure 8. Representative flow cytometry plots of IL-8$^+$ naive T cells in patients with thymoma recurrence at different time points. (a) The representative flow cytometry plots of IL-8$^+$CD31$^+$ cells in naïve CD4$^+$ T cells (upper panel) or naïve CD8$^+$ T cells (lower panel) in PBMCs at different time points before and after thymoma resection in Case #1 patient with thymoma recurrence. (b) The representative flow cytometry plots of IL-8$^+$CD31$^+$ cells in naïve CD4$^+$ T cells (upper panel) or naïve CD8$^+$ T cells (lower panel) in PBMCs at different time points before and after thymoma resection in Case #2 patient with thymoma recurrence. (c) The representative flow cytometry plots of IL-8$^+$CD31$^+$ cells in naïve CD4$^+$ T cells (upper panel) or naïve CD8$^+$ T cells (lower panel) in PBMCs at different time points before and after the first thymoma resection in Case #3 patient with thymoma recurrence. (d) The representative flow cytometry plots of IL-8$^+$CD31$^+$ cells in naïve CD4$^+$ T cells (upper panel) or naïve CD8$^+$ T cells (lower panel) in PBMCs at different time points before and after the first thymoma resection in Case #4 patient with thymoma recurrence.

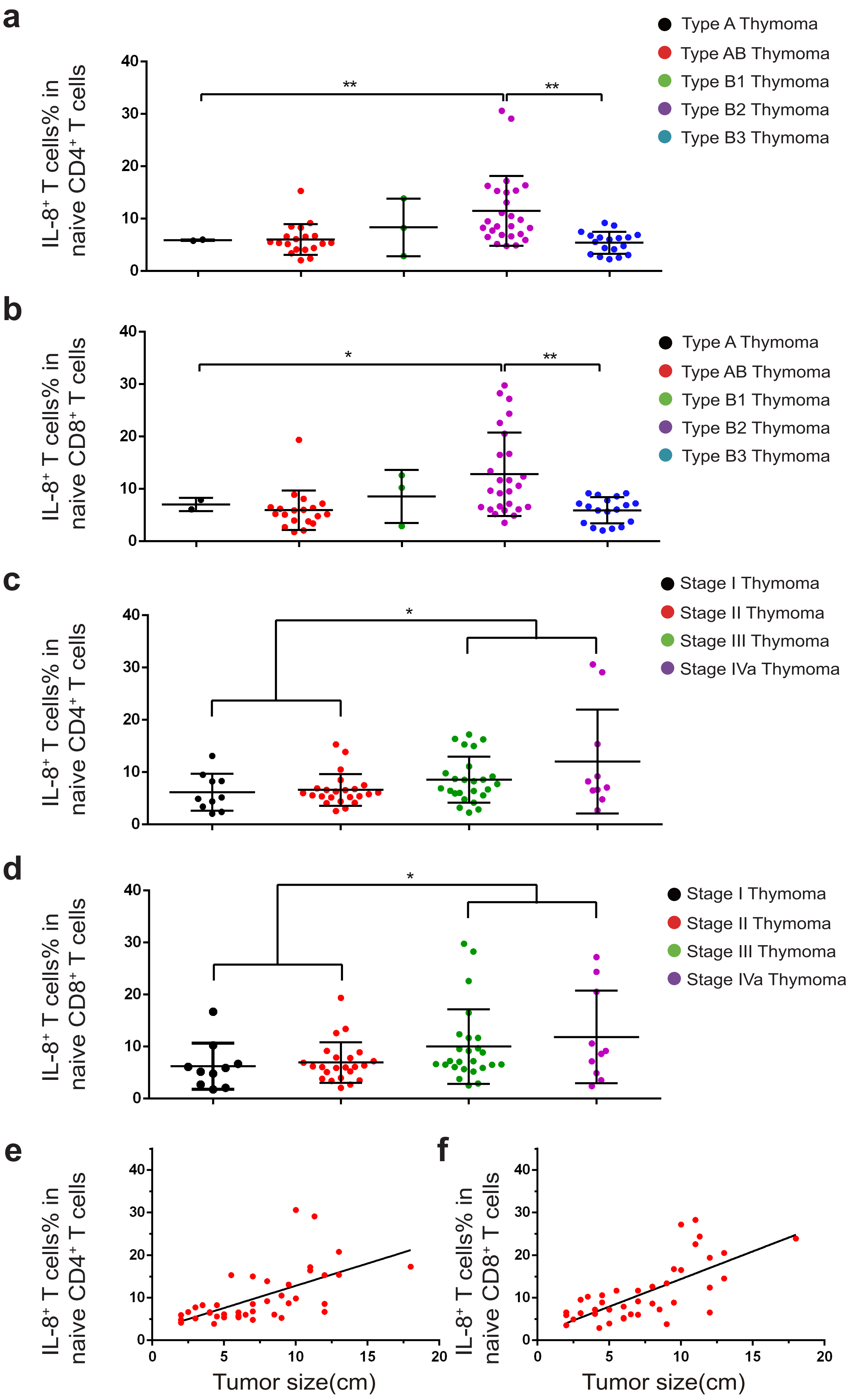
a
Type A Thymoma
Type AB Thymoma
Type B1 Thymoma
Type B2 Thymoma
Type B3 Thymoma
IL-8⁺ T cells% in naive CD4⁺ T cells
**
**
b
Type A Thymoma
Type AB Thymoma
Type B1 Thymoma
Type B2 Thymoma
Type B3 Thymoma
IL-8⁺ T cells% in naive CD8⁺ T cells
*
**
c
Stage I Thymoma
Stage II Thymoma
Stage III Thymoma
Stage IVa Thymoma
IL-8⁺ T cells% in naive CD4⁺ T cells
*
d
Stage I Thymoma
Stage II Thymoma
Stage III Thymoma
Stage IVa Thymoma
IL-8⁺ T cells% in naive CD8⁺ T cells
*
e
IL-8⁺ T cells% in naive CD4⁺ T cells
Tumor size(cm)
f
IL-8⁺ T cells% in naive CD8⁺ T cells
Tumor size(cm)

Supplementary Figure 9. The variability of IL-8$^+$ naïve T cells within thymoma patients is caused by the variability of clinical pathological features. (a) The frequencies of IL-8$^+$CD4$^+$ naïve T cells in PBMCs from patients with WHO subtype A, AB, B1, B2 or B3 thymomas. (b) The frequencies of IL-8$^+$CD8$^+$ naïve T cells in PBMCs from patients with WHO subtype A, AB, B1, B2 or B3 thymomas. (c) The frequencies of IL-8$^+$CD4$^+$ naïve T cells in PBMCs from thymoma patients at Masaoka stage I, II, III or IVa. (d) The frequencies of IL-8$^+$CD8$^+$ naïve T cells in PBMCs from thymoma patients at Masaoka stage I, II, III or IVa. (e) The relationship between IL-8$^+$CD4$^+$ naïve T cells and tumor sizes. (f) The relationship between IL-8$^+$CD8$^+$ naïve T cells and tumor sizes. The summary data in (a-d) were presented as mean ± SD. Statistical differences were determined by two-sided Krusal-Wallis Analysis of Variance test and adjusted with the Benjamini–Hochberg procedure. Adjusted P values were indicated by * ($p<0.05$), or ** ($p<0.01$). Source data are provided as a Source Data file.

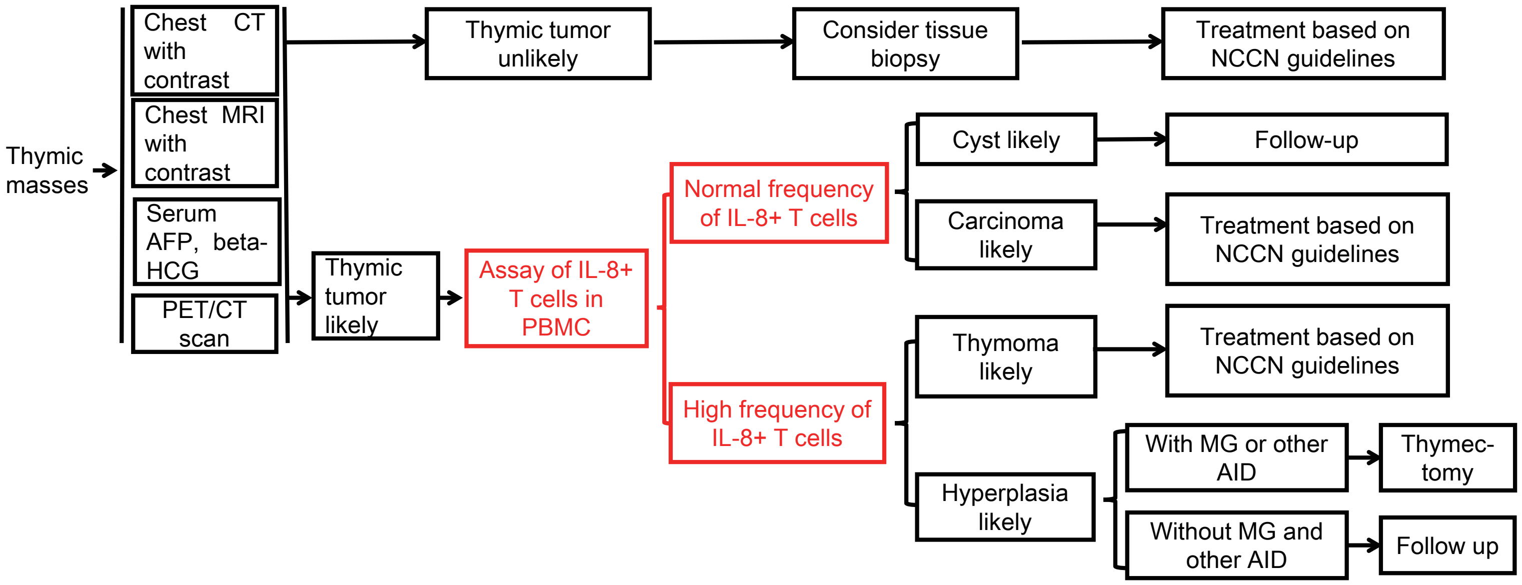

Supplementary Figure 10. A proposal for the diagnostic procedure for thymic masses based on CT scans/MRIs and IL-8 detection. A revision of NCCN guideline was proposed. The major change in the proposed guideline is the recommendation to apply “the IL-8 evaluation in T cells” to the differential diagnosis of thymic masses. By combining chest CTs/MRIs with contrast with the detection of IL-8, most thymic masses could be accurately identified. Red color highlights the IL-8 assays in the proposed NCCN guideline.

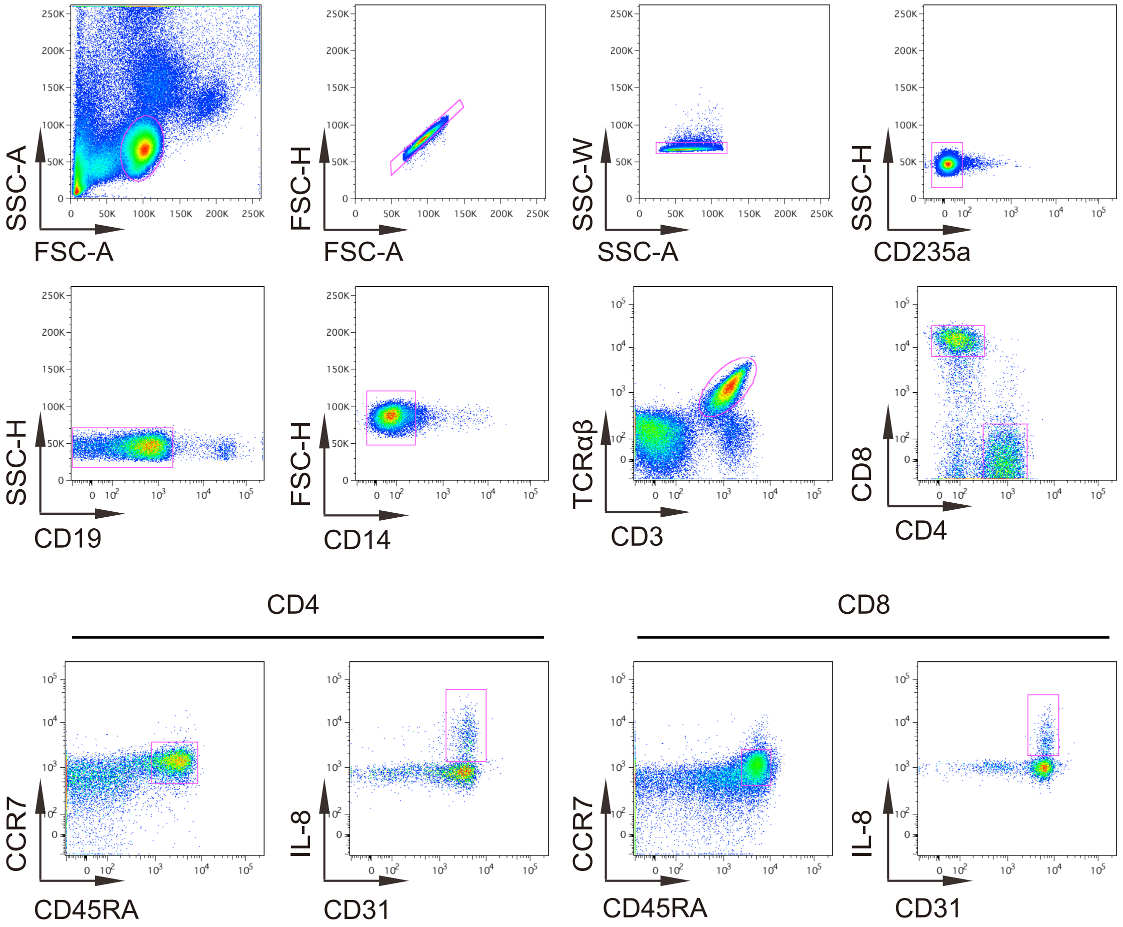

Supplementary Figure 11. Gating strategy of IL-8$^+$ naïve T cells in a representative thymoma case. IL-8$^+$CD4$^+$ naïve T cells were gated as CD235a$^-$CD19$^-$CD14$^-$CD3$^+$TCRαβ$^+$CD4$^+$CD8$^-$CD45RA$^+$CCR7$^+$CD31$^+$IL-8$^+$. IL-8$^+$CD8$^+$ naïve T cells were gated as CD235a$^-$CD19$^-$CD14$^-$CD3$^+$TCRαβ$^+$CD4$^-$CD8$^+$CD45RA$^+$CCR7$^+$CD31$^+$IL-8$^+$. CD14 staining is to exclude the interference of macrophages.
