# Supplementary material for: Interleukin-8 as a candidate for thymoma identification and recurrence surveillance
Source: Nat Commun. 2020 Sep 28;11:4881. doi: 10.1038/s41467-020-18697-x (PMC7522267; doi:10.1038/s41467-020-18697-x)
Supplement: Supplementary file 3 — Reporting Summary [file 41467_2020_18697_MOESM3_ESM.pdf]

nature research

Corresponding author(s): Jianyong Ding

Last updated by author(s): Aug 24, 2020

Reporting Summary

Nature Research wishes to improve the reproducibility of the work that we publish. This form provides structure for consistency and transparency in reporting. For further information on Nature Research policies, see our Editorial Policies and the Editorial Policy Checklist.

Statistics

For all statistical analyses, confirm that the following items are present in the figure legend, table legend, main text, or Methods section.

n/a	Confirmed	
☐	☒	The exact sample size (*n*) for each experimental group/condition, given as a discrete number and unit of measurement
☐	☒	A statement on whether measurements were taken from distinct samples or whether the same sample was measured repeatedly
☐	☒	The statistical test(s) used AND whether they are one- or two-sided<br>*Only common tests should be described solely by name; describe more complex techniques in the Methods section.*
☐	☒	A description of all covariates tested
☐	☒	A description of any assumptions or corrections, such as tests of normality and adjustment for multiple comparisons
☐	☒	A full description of the statistical parameters including central tendency (e.g. means) or other basic estimates (e.g. regression coefficient) AND variation (e.g. standard deviation) or associated estimates of uncertainty (e.g. confidence intervals)
☐	☒	For null hypothesis testing, the test statistic (e.g. *F*, *t*, *r*) with confidence intervals, effect sizes, degrees of freedom and *P* value noted<br>*Give P values as exact values whenever suitable.*
☒	☐	For Bayesian analysis, information on the choice of priors and Markov chain Monte Carlo settings
☐	☒	For hierarchical and complex designs, identification of the appropriate level for tests and full reporting of outcomes
☐	☒	Estimates of effect sizes (e.g. Cohen's *d*, Pearson's *r*), indicating how they were calculated

Our web collection on statistics for biologists contains articles on many of the points above.

Software and code

Policy information about availability of computer code

Data collection	No software was used for data collection.
Data analysis	R, Graphpad version 6.0, SPSS Statistics version 19; Flowjo 8.8.7

For manuscripts utilizing custom algorithms or software that are central to the research but not yet described in published literature, software must be made available to editors and reviewers. We strongly encourage code deposition in a community repository (e.g. GitHub). See the Nature Research guidelines for submitting code & software for further information.

Data

Policy information about availability of data

All manuscripts must include a data availability statement. This statement should provide the following information, where applicable:
- Accession codes, unique identifiers, or web links for publicly available datasets
- A list of figures that have associated raw data
- A description of any restrictions on data availability

The authors declare that the data supporting the findings of this study are available within the article and its supplementary information files.

Field-specific reporting

Life sciences study design

All studies must disclose on these points even when the disclosure is negative.

Sample size	Described in "METHODS/Study design": All patients enrolled in this study underwent surgical resection for thymic masses between December 2015 and May 2020 in the Thoracic Surgery Department, Zhongshan Hospital, Fudan University (Shanghai, China) and Xuhui Central Hospital (Shanghai, China). Healthy controls were collected from the Physical Examination Center, Zhongshan Hospital (Shanghai, China). Samples were divided into two cohorts: discovery set contains 40 samples (30 patients and 10 healthy controls) while validation set contains 186 samples (153 patients and 33 healthy controls)<br>In order to determine sample size, we performed the sample size and power calculations by using R. Based on the results from discovery set, assuming the AUC is 0.95, a sample size of 12 (case: 6, control: 6) is needed to achieve 95% power at 5% significance level. In contrast, in the validation study, with 186 cases in ROC calculation the power for detecting an AUC of 0.95 is 100%. Therefore, current sample size is enough for the study objective.
Data exclusions	No data was excluded in our investigation.
Replication	For IL-8 detection, replicate experiments were successfully performed across multiple patients. For Trecs Analysis, assays were repeated in triplicate.
Randomization	The discovery and validation cohorts were randomly divided.
Blinding	The collection of clinical information and flow cytometry analysis in the validation set were blindly performed by different members.

Reporting for specific materials, systems and methods

We require information from authors about some types of materials, experimental systems and methods used in many studies. Here, indicate whether each material, system or method listed is relevant to your study. If you are not sure if a list item applies to your research, read the appropriate section before selecting a response.

Materials & experimental systems

n/a	Involved in the study	
☐	☒	Antibodies
☒	☐	Eukaryotic cell lines
☒	☐	Palaeontology and archaeology
☒	☐	Animals and other organisms
☐	☒	Human research participants
☒	☐	Clinical data
☒	☐	Dual use research of concern

Methods

n/a	Involved in the study	
☒	☐	ChIP-seq
☐	☒	Flow cytometry
☒	☐	MRI-based neuroimaging

Antibodies

Antibodies used	Antibodies used in this study:<br>Antigen Fluorophor Clone Catalogue Provider<br>CD15 eFluor 450 MMA 43-0158-42 eBioscience<br>TCRα β APC IP26 17-9986-42 eBioscience<br>CD3 Alexa Fluor 700 UCHT 557943 Biolegend<br>CD45RA Bv785 HI100 304140 Biolegend<br>CCR7 PECy7 G043H7 353226 Biolegend<br>IL-8 FITC E8N1 554720 Biolegend<br>CD31 PerCP/eFluor 71 0 UVM59 46-0319-42 Invitrogen<br>CD14 APCCy7 M5E2 301819 Biolegend<br>CD8 Bv650 RPA-T8 301041 Biolegend<br>CD4 Bv605 SK3 63-0047-42 eBioscience<br>CR2 PE BU32 354903 Biolegend<br>PTK7-biotin Poly-clonal generated by AbMART
Validation	All commercial antibodies were validated by their respective manufacturers. For anti-PTK7 antibody, the specificity of the antibody has been validated by staining 293T overexpressing with PTK7.

Human research participants

Policy information about studies involving human research participants

Population characteristics	Described in "Table .1".
Recruitment	Heparinized blood samples, tumor tissues and paratumor tissues were collected from patients who were admitted to Zhongshan Hospital and Xuhui Central Hospital. Healthy blood samples were collected from volunteers who were recruited to Zhongshan Hospital.Bias have been reduced to mininal since samples from healthy blood donors were randomly allocated to the study and no data on age, sex or any other population were available during experiments.<br>characteristic is available.
Ethics oversight	Described in "METHODS/Study design": This study was approved by the Zhongshan Hospital Research Ethics Committee and the Research Ethics Committee of Xuhui Central Hospital. Ethical approval was obtained from the Zhongshan Hospital Research Ethics Committee. Written informed consent was obtained from each patient. All specimens were handled and made anonymous according to the ethical and legal standards.

Note that full information on the approval of the study protocol must also be provided in the manuscript.

Flow Cytometry

Plots

Confirm that:

- [x] The axis labels state the marker and fluorochrome used (e.g. CD4-FITC).
- [x] The axis scales are clearly visible. Include numbers along axes only for bottom left plot of group (a 'group' is an analysis of identical markers).
- [x] All plots are contour plots with outliers or pseudocolor plots.
- [x] A numerical value for number of cells or percentage (with statistics) is provided.

Methodology

Sample preparation	PBMCs were isolated from heparinized blood samples using Lymphoprep (Stem Cell Technologies #07861) density gradient centrifugation at 500g for 30 min at room temperature. PBMCs were then washed twice with T cell medium (Dulbecco's Modified Eagle's Medium (DMEM) containing 10% heat-inactivated fetal bovine serum (FBS), 2 mM L-glutamine, penicillin-streptomycin, nonessential amino acids, sodium pyruvate, vitamins, 10 mM HEPES and 50 μM 2-mercaptoethanol), and resuspended in T cell medium.<br>For thymocytes isolation, thymus specimens including thymic tumor tissues and paratumor tissues were kept on ice after surgical removal. Visible blood vessels were removed and tissues were washed with ice-cold DMEM medium. Tissues were minced by sterilized scissors, and meshed in a sterile 70-μm cell strainer. Then, the strainer was rinsed with 5 ml cold DMEM medium, and the single cell suspension was filtered again using a 70-μm cell strainer to remove large debris. Cells were washed twice with cold DMEM medium, and re-suspended with T cell medium.
Instrument	BD Fortessa, BD FACS Aria II
Software	FACSDiva for data acquisition and FlowJo 8.8.7 for post-acquisition analyzes.
Cell population abundance	The final population we showed were collected at least 3,000 events.
Gating strategy	Cell debris were excluded by SSC-A and FSC-A gating (G1), Doublets were excluded by FSC-A and FSC-H gating (G2) and SSC-A and SSC-W(G3) for all flow cytometry analysis. To eliminate the interference of TCR positive myeloid cells on IL-8 measurements, myeloid cells were excluded by gating CD14-(G4) and CD15-(G5) subsets. CD31+ RTEs were gated by CD3 +TCR+CD4+CD45RA+CCR7+CD31+ subsets. IL-8 positive naive T cells were gated by TCR+CD3+CD8-CD45RA+CCR7+CD31+IL-8 + (IL-8 positive CD4+ naive T cells) or TCR+CD3+CD8+CD45RA+CCR7+CD31+IL-8+ (IL-8 positive CD8+ naive T cells) subsets. CR2+ RTEs were gated by CD3+TCR+CD4+CD45RA+CCR7+CD31+CD25-CR2+ subsets. PTK7+ RTEs were gated by CD3+TCR +CD4+CD45RA+CCR7+CD31+PTK7+ subsets.

- [x] Tick this box to confirm that a figure exemplifying the gating strategy is provided in the Supplementary Information.
